# Understanding contexts: how explanatory theories can help

**DOI:** 10.1186/s13012-019-0872-8

**Published:** 2019-03-06

**Authors:** Frank Davidoff

**Affiliations:** 1 Lexington, USA; 2 Geisel School of Medicine, Dartmouth College, Hanover, NH 03755 USA

## Abstract

**Objective:**

To rethink the nature and roles of context in ways that help improvers implement effective, sustained improvement interventions in healthcare quality and safety.

**Design:**

Critical analysis of existing concepts of context; synthesis of those concepts into a framework for the construction of explanatory theories of human environments, including healthcare systems.

**Data sources:**

Published literature in improvement science, as well as in social, organization, and management sciences. Relevant content was sought by iteratively building searches from reference lists in relevant documents.

**Results:**

Scientific thought is represented in both causal and explanatory theories. Explanatory theories are multi-variable constructs used to make sense of complex events and situations; they include basic operating principles of explanation, most importantly: transferring new meaning to complex and confusing phenomena; separating out individual components of an event or situation; unifying the components into a coherent construct (model); and adapting that construct to fit its intended uses. Contexts of human activities can be usefully represented as explanatory theories of peoples’ environments; they are valuable to the extent they can be translated into practical changes in behaviors.

Healthcare systems are among the most complex human environments known. Although no single explanatory theory adequately represents those environments, multiple mature theories of human action, taken together, can usually make sense of them. Current mature theories of context include *static models*, *universal-plus-variable models*, *activity theory and related models*, and the *FITT framework* (Fit between Individuals, Tasks, and Technologies). Explanatory theories represent contexts most effectively when they include basic explanatory principles.

**Conclusions:**

Healthcare systems can usefully be represented in explanatory theories. Improvement interventions in healthcare quality and safety are most likely to bring about intended and sustained changes when improvers use explanatory theories to align interventions with the host systems into which they are being introduced.

## Introduction

Human contexts—defined in this commentary primarily as the meaning of human environments to the people who live and work in them—are major determinants of the effectiveness and generalizability of interventions to improve healthcare quality and safety [1, 2]. Despite the importance of contex, much about it remains obscure, as do the specific mechanisms by which local contexts affect the implementation of improvement interventions. As a consequence, context is still sometimes vaguely referred to in scholarly work as “All those things in the situation which are relevant to meaning in some sense, but which I haven’t identified.”([2] p. 6).

Context plays an important role in both improvement science and implementation science; limited understanding of context therefore limits understanding of both the fundamental principles of improvement and the actions that put improvements into practice. Achieving deep understanding of context is a challenge that has baffled serious improvers, researchers, and scholars for years [2]. This difficulty [3] suggests that multiple complementary explanatory theories might prove more useful than any single theory in understanding both context in general and specific local contexts.

This commentary is intended as a complement to the SQUIRE guidelines for publication of work in quality improvement [4]. It explores the premise that explanatory theories of human environments can help improvers work flexibly from first principles rather than rigid formulas, and, as is true for good theories generally [6], can provide improvers with explicit reasons why particular interventions are likely to be effective in specific systems; it examines the nature of explanatory theories and the basic principles of explanation, considers the contributions of those principles to mature (i.e., fully-developed, refined) explanatory theories of complex human environments, and considers the nature of the data needed in constructing explanatory theories of local environments, and the methods used for gathering the requisite data. The commentary proposes, finally, that it is both appropriate and useful early in the planning of an improvement program, to create an explanatory theory of the local healthcare environment into which planned intervention is to be introduced, then use that theory in linking the intervention with that environment. The commentary also encourages improvers to reconsider and revise the initial explanatory theories from time to time as more is learned about the local environment during the improvement process.

### Explanatory theories

Scientific thought is built primarily around two complementary mental constructs [5]: causal and explanatory theories. Explanatory theories are created to help to understand complex, confusing events and situations; they often also serve as sources of testable causal theories of events and situations.

Although explanatory theories are sometimes thought to play a less central role in science than causal theories [5–7], many explanatory theories— including the theory of evolution, the periodic table of the elements, and the structure of DNA—have proven uniquely helpful in understanding important phenomena in natural sciences. Political science is built largely around explanatory theories [7]; process flow diagrams and Pareto charts are among the explanatory theories that help understand events and situations in improvement science [8].

## Methods

The concepts in this commentary were developed from the published literature in improvement science as well as the social, behavioral, organizational, improvement, and management sciences. Sources that proved especially important include Bate et al. [2] on the dynamic properties of context, Squires et al. [3] on the construction of explanatory theories, Braithwaite et al. [9] and Greenhalgh [10] on complexity, Nardi [11] and Greenhalgh et al. [12] on theories of human action, Vandenbroucke [5] and Clarke and Primo [7] on explanatory theory, and Pitt [13] on the fundamentals of explanation. Literature searches were built out from reference citations in these and related publications.

The author’s experience as editor of a major clinical journal (*Annals of Internal Medicine*), and as publications editor at the Institute for Healthcare Improvement (Cambridge, MA), also helped in constructing this commentary. Discussions in the improvement science development group at the Health Foundation (London, UK) and in the Standards for Quality Improvement Reporting Excellence (SQUIRE) leadership group [4] also contributed importantly to this effort.

## Results

### The complexity and dynamism of human environments

The most salient properties of human environments are arguably their *complexity* [9, 10] and their *dynamic nature* [2]. This commentary rests on the concept of “complex systems” summarized in Table 1.

**Table 1:** Distinctive properties of complex systems (adapted from references [9, 10, 14])

Property	Descriptor
Prominent properties	
Self-organization	The coming together of individual agents in the absence of central control to form new organizational programs and structures
Non-linear behaviors	Large inputs result in small changes in output and vice versa
Co-evolution	Organizational behaviors change in response to environmental changes
Emergent properties	Qualitatively new phenomena, including structures, capabilities, and behaviors, that arise unpredictably from the interaction of independent agents
Subtle properties	
Boundaries	Internal and external boundaries between domains and decision-making levels are often “fuzzy”
Rules	Many actions are guided in practice by internalized rules that are informal and not consciously recognized
Subsystems	Small subsystems are commonly embedded within larger, more complex systems
Tensions	Internal tensions among agents and groups are inherent, i.e., they cannot be eliminated but can be managed

The degrees of complexity in human systems are usefully characterized in the following schema [14], in which the cooking of a specific dish is represented as *simple*. Challenges at this basic level are usually managed successfully by following explicit, straightforward recipes or protocols.

By comparison, sending a rocket to the moon is *complicated* for multiple psychological, social, and technical reasons. Successful management of complicated challenges often requires the use of dedicated management tools such as checklists (mainly to overcome the limitations of human memory) and protocols that map out contingency-dependent decision points (mainly to avoid oversimplification).

Finally, the challenge of raising a child can be seen as *complex*, largely because it involves such a large number of variables, many of them poorly defined, which often leads to unpredictable outcomes, e.g., when the experience of raising one child successfully is of little use in raising the next.

### Principles of explanation (sense-making)

Although a human event or situation can sometimes be explained adequately in terms of causal mechanisms, the inherent complexity and dynamic nature of events and situations usually requires explanations that go beyond causality and include descriptive explanatory principles [5, 6, 10, 13–16]. Most importantly, those principles include transferring new meaning to the event or situation, establishing its familiarity and internal logic, separating out its individual components, unifying its components into a coherent mental construct or “gestalt”, and adapting the explanation to fit its intended uses.

#### Transferring (sharing) meaning

The classic human system for transferring or sharing meaning is, of course, language [17]: witness the substantial loss or distortion of its meaning that results when a word or phrase is taken out of context, and conversely the greater precision of a literature search that uses search terms embedded in linguistic contexts, when contrasted with a search that uses search terms lacking such embellishment [18, 19]. (Salmon proposes that the transfer of information, energy or causal inference between *processes* is more meaningful than transfer between *events* [16].)

#### Familiarity

Familiarity, by itself, is neither necessary nor sufficient to make sense of an event or situation. But familiarity is nonetheless an important component of explanation, because a sense of familiarity provides a sense of understanding ([20], p. 52). Metaphor is often the chosen mechanism for transferring meaning from familiar things to those that are less familiar, a property that prompted Aristotle to comment that it is metaphor that most produces knowledge. The psychologist Julian Jaynes has argued that metaphor is not a “mere extra trick of language” but is rather “the very constitutive ground of language,” and that “it is by metaphor that language grows” ([20], pp. 48-9).

#### Logic

Explanation in natural sciences is usually considered adequate when its logic is clear, as when statement of a general law (a “regularity”) is coupled with statement of a specific antecedent condition. In physics, for example, a statement such as “All wave phenomena of a certain type satisfy the law of refraction, and light is a wave of that type” is accepted as a logical construct that meaningfully explains the refraction of light ([13] p. 10]).

#### Separating out and unifying components

By themselves, the individual components of an event or situation ordinarily have little if any inherent meaning. But the construct that results when those components are brought together to make a coherent whole (usually as a narrative, map, model, or mathematical expression) is uniquely helpful in making sense of that event or situation [4, 21, 22]. Important new meanings can emerge as well—often unexpectedly—from the resulting construct. For these reasons, some philosophers of science consider unification of a phenomenon’s individual components into a coherent whole as the main principle by which explanation renders a phenomenon understandable [4, 5, 21, 22].

The sharing of meaning among a phenomenon’s individual components finds expression in catch-phrases such as the *jigsaw puzzle effect*, and “The whole is greater than the sum of its parts.” On a more grand scale, the theory of evolution is said to acquire its explanatory power when “an apparently modest allegiance to mere fact gathering” abruptly crystallizes into a “whole world view” [23].

Details of the mental process through which unification creates explanations unfortunately remain obscure. And curiously, even a highly coherent construct of an event or situation does not necessarily help understand whether its components are truly independent, whether the interactions among them are uni-directional or recursive, and which components (if any) are most important in determining its overall behavior. Moreover, craftspeople such as watchmakers and car mechanics understand that success in their work depends on their ability to separate out the components of the complex systems they are called on to assemble or repair (disaggregate them) at least as much as on their ability to understand how the components contribute collectively to an event or situation’s overall behavior (unify them). At least in theory, the explanatory principles of disaggregation and unification appear to contradict each other, but in practice, the two principles are often complementary. In managing a human system, for example, a leader’s ability to unify various groups’ individual modes of decision-making can complement his or her ability to distinguish those modes from one another [24].

#### Adapting explanations

Explanatory theories are arguably successful to the extent people can translate them into practical implementation behavior—e.g., manage the environments in which they live and work or predict the likelihood that a specific event will happen in the future ([16] p. 77). Not surprisingly, therefore, the explanatory theories people develop on their own to manage their personal environments differ substantially from the ones they develop collectively to further the work of the organizations in which they work. For similar reasons, personal and organization-related explanatory theories differ from those that outside researchers create to understand these various environments.

##### Personal contexts

Peoples’ intense, universal need to give meaning to “the brutal aboriginal flux” of their lived experience [1] suggests that humans can be defined as “reason-giving animals” [25]. They begin creating explanatory theories of their personal environments at an extremely early age [26], then extend and refine those theories as they and their personal environments change over time. Personal explanatory theories are usually implicit and poorly articulated; they can also be distorted, incomplete, or inappropriate since they frequently lack independent reality testing.

##### Organizational and professional contexts

Workers in organizations are called on to create explanatory models that make sense of the internal structure and function of those organizations, as well as of the external environments in which their organizations are embedded. Weick et al. describe this work as a creative, collaborative undertaking that involves “language, talk, and communication” and is “ongoing, subtle, swift, social, and easily taken for granted” [1, 27, 28]. Early in this sense-making process, workers in an organization “bracket” information (i.e., identify items they see as especially relevant to their particular situation), then name (label) those items, which stabilizes the streaming of their experience [1].

The way people in organizations envision events and situations also immediately begins their social and administrative work of organizing, because bracketing and labeling events predisposes them to find common ground and provides them with a set of cognitive categories, plus a typology of potential actions. (Bracketing central venous catheter infection and labeling it as primarily a *social* rather than a *biological* problem [29] played an important role in shaping an intervention that successfully lowers the infection rate [30].) Workers then use such newly defined contextual elements as they literally talk their organization-related explanatory theories into existence [1].

The sense-making process described above closely resembles the one that professionals in applied disciplines, together with their clients, use to make sense of the problem situations they are called on to manage ([31], pp. 267–83). More specifically, medical professionals will recognize its resemblance to the process by which they and their patients formulate the essential explanatory theories they know as *diagnoses*.

### Mature explanatory theories of human environments

People initially sketch out rough explanatory theories of environments which usually involve basic principles of explanation, then subsequently broaden and refine these nascent constructs into more *mature* theories. Important examples of such mature explanatory theories include *static theories*, *universal-plus-variable theories*, *activity theory* and *related general theories of human action*, and the *FITT framework* (Fit between Individuals, Task, and Technology).

#### Static theories

Several research groups have developed explanatory theories of outstanding healthcare systems by selecting the components they judge to be most closely associated with certain systems’ ability to deliver exceptionally safe, high-quality care [32–36], then assembling those components into structured models. (A recent international effort is engaged in constructing a new and more meaningful theory of this type [3]).

The individual components identified in these theory-building exercises—buildings, equipment, leadership, geographic location, teaching status, financial and intellectual resources, and the like—are quite heterogeneous and the resulting constructs often pay little attention to functional relationships among the components or to the ways in which the process of care plays out over time for individual patients. Metaphorically speaking, then, explanatory theories such as these describe the *anatomy* of exceptional healthcare environments, but not their *physiology*; that is, they are *static*, which could account for the limited ability of this type of explanatory theory to explain variation in the effectiveness of improvement interventions across different healthcare systems.

#### Universal-plus-variable models

Working from detailed on-site observations in high-performing healthcare systems, Bate et al. [2, 37] have constructed a generalized explanatory theory of such systems. Their experience is reflected in their comment that “although research has provided an abundance of data on key success factors in QI efforts, very little was previously known about how these combine and interact with each other in the improvement process over time.” They comment further that the context of a healthcare system is “a process; dynamic, fluid, and constantly moving, not lumpen, material, or static,” and that “it is the dynamic and ongoing interaction between [the domains of an environment] rather than any one of them individually or independently, that accounts for the effectiveness of a QI intervention,” as well as for “the striking variation between similar QI interventions in different places” ([2]p. 11).

These investigators then refine and sharpen the focus of their emerging explanatory theory by postulating that a healthcare system’s ability to deliver outstanding care lies in the combination of the two major components—*universals* and *variables*—that characterize an organization’s local situation. More specifically, they identify the challenges inherent in several distinct areas—physical/technological, emotional, educational, cultural and political, and structural—as the *universals* in *all* healthcare organizations; they also characterize the actions that individual workers and groups take in response to those challenges as differing both *within* and *across* organizations to the point where those actions and the possible combinations among them can be assumed to be “practically innumerable” ([37], p. 168), i.e., they are the *variables*.

The resulting universal-plus-variable explanatory theory of human contexts gains plausibility from its affinity with other established cognitive systems in which people represent the complex meanings that matter to them. The best known and arguably most important of such systems is of course language [17]; people produce language by embedding differing strings of individual words (the variables) in a relatively small number of stable grammatical structures (the universals). They then use the resulting construct to create a virtually unlimited number of statements that are meaningful to others, even though many of those statements have not been seen or heard previously.

Music provides another illuminating example of a meaningful universal-plus-variable explanatory theory [39]. Composers in each musical tradition embed differing arrays of tones (variables) in a limited set of stable, widely recognized harmonic constructs (universals). One critic has elegantly captured this explanatory theory of music (or at least of Western music) in his pithy comment that “Mozart used the same B-flat as everyone else.”

#### Activity theory and related models of human action

The universal-plus-variable explanatory theory of contexts also resonates with several earlier mature explanatory theories of human action, including Activity Theory and related models [11]. Some of these action theories are now seen as especially useful in understanding the interaction between people and computer systems [12]. In these theories, it is precisely the ongoing bi-directional interaction between static human environments and the dynamic needs, interests, and experiences people bring to encounters with those environments that creates most of the contexts (meanings) of human life. For example, context is understood as follows in Activity Theory as an overarching, albeit secondary, consideration: “[W]hat takes place in an activity system composed of object, actions, and operations, *is* the context… [C]ontext is not an outer container or shell inside of which people behave in certain ways.” Context in these theories is thus “both internal to people…and at the same time, external to people” [11], i.e., as an integrated whole. This unifying perspective invalidates “simplistic explanations that divide internal and external, and schemes that see context as external to people.”

#### The FITT framework (Fit between Individuals, Tasks, and Technologies)

Developed largely to explain the adoption of information and communication technologies (IT) [40], the FITT framework clearly distinguishes an organization’s established and widely recognized tasks and technologies from its workers’ shifting dynamic behaviors [5, 12, 40, 41] (Table 2), and in that respect, it resembles other universal-plus-variable explanatory theories of human activity.

**Table 2:** Use of explanatory principles in constructing an electronic decision-support system to improve postoperative care (adapted from references [5, 6, 13, 41])

Explanatory principle	Use of the principle
Transfer/sharing of meaning	*External knowledge* from published literature on clinical guidelines, existing protocols from academic affiliates in the VA system, and discussions with subject matter experts was brought together with *internal knowledge* about the baseline strengths and limitations of a specific local VA hospital’s environment and its processes of care
Familiarity	Observed performance (i.e., proportion of prophylaxis-eligible patients whose orders for postoperative care were selected from the new electronic menu) improved demonstrably as staff familiarity with the system increased over time
Logic	Empirical evidence that anti-thrombosis regimens effectively reduce risk of postoperative thromboembolism (the regularity) was coupled with the increased thromboembolic risk that existed locally in eligible individual postoperative patients (the antecedent condition)
Separating out environmental components	The contributions of individuals, tasks, and technologies to an effective DVT prophylaxis strategy were re-examined iteratively during implementation of the emerging electronic decision support system
Unifying components of the environment	The following major components were unified into a coherent model of the local environment that improvers used in implementing the new postoperative DVT prophylaxis strategy: *Individuals*: surgical residents, who wrote most of the post-operative orders; *Task*: choosing and ordering clinical interventions appropriate for each patient’s degree of thromboembolic risk; *Technology*: electronic order menus embedded in a pre-existing electronic medical record
Adaptation to intended uses	Order menus were modified in response to the results of extensive repeated multilevel user testing; project staff (i.e., surgical residents, pharmacists, and nurses) also received education on the purpose and design of the new order system

As noted elsewhere [6], the FITT framework has been used to guide the successful implementation of an innovative electronic order system for post-operative surgical care [41]. Researchers in that study explicitly used the FITT framework to help them interweave their new electronic system with the healthcare environment in which they implemented it.

### The nature of data needed to construct explanatory theories of healthcare environments

Adequate understanding of human environments requires that explanatory theories take the enormous complexity of those environments appropriately into account. Although complexity of this magnitude can be a cause for despair among improvers and researchers, the statistician George Box’s pungent comment that “All theories are wrong, but some are illuminating and useful” offers reassurance that creating explanatory theories of human environments, including healthcare systems, is likely on balance to be worth the effort.

#### Data used to create meaningful explanatory theories of human environments

Creating explanatory theories of human environments that help implement successful improvement interventions apparently requires open-ended, multi-level data on working relationships in organizations [1, 9–11, 29, 31, 36–38, 41–48]. Research groups are now laboring to clarify the essential nature of such data (Table 3), while also obtaining insights into effective techniques for collecting and analyzing those data (Table 4).

**Table 3:** Characteristics of data that contribute meaningfully to explanatory theories of human environments (adapted from references [9, 42–48])

Characteristic	Descriptor
Trust	Willingness of a person to be vulnerable to another person
Mindfulness	Openness to new ideas and different perspectives; fully engaged presence; rich, discriminating awareness; seeking novelty, even in routine situations
Heedfulness	Interactions in which individual people are sensitive to both their individual, narrowly focused tasks, and the way those roles and actions affect the roles and actions of the whole group
Respectful interaction	Honest, self-confident, appreciative interactions among individual agents—these often create new meaning
Diversity	A collective cognitive property: one that supports moderate differences in individual perspectives, thoughts, and views of the world, to enhance group problem-solving and creativity
Social and task relatedness	Maintaining a balanced combination of work-related and personal aspects of care delivery, which can help staff provide care characterized by community, connectivity, and intimacy
Effective (rich/lean) communication	Using the mode of communication most appropriate to each situation: • Rich: Face-to-face mode is best used when messages/issues are uncertain and/or ambiguous; • Lean: Mass documents, numeric formats are best used for clearly defined, non-threatening issues

**Table 4:** Methods for collecting and analyzing data that help to plan, implement, and evaluate the impact of improvement interventions (adapted from references [31, 42, 47, 48])

Directly observing professional practices during work activities	
Interviewing (in depth) patients and families, physicians, and other key staff	
Obtaining surveys of staff, structured checklists of practice environments, and medical chart reviews	
Systematically identifying and validating case narratives of connections between professional process elements or solutions	
Creating process-oriented narratives and maps that go beyond technical aspects of interventions to represent properties of the professional communities into which interventions are introduced; keeping records of the dynamic cultural and political changes (including both events and structures) that appear to underlie observed changes in clinical processes and outcomes	
Collecting information from staff-generated journals and field notes that record the practice characteristics, events, and situations seen as affecting the observed range of success in practice improvements	
Using complex adaptive systems theory in data analysis	
Recording examples of emergent properties, self-organization, and co-evolution within the organizational environment	

It is important to note in this connection that improvement interventions reach their full potential more successfully when their implementation builds on the complexity of the systems they intend to change than when they underestimate or ignore that complexity [9]. Even documenting that a healthcare system has “a long way to go” to achieve specific solutions within each of the six universal challenge area (in contrast to being either “some way there” or “already there”) can help improvers pinpoint current gaps and opportunities in that system’s quality and safety, and facilitate productive discussions on their future improvement efforts (Cf. Codebook for Quality Improvement Practice, for example) ([37], p., 177).

In like fashion, answering a question regarding organizational complexity (e.g., “How did this practice miss a diagnosis?”) can be more effective in changing system performance than obtaining answering a narrowly focused question such as “How did an individual practitioner miss a diagnosis?”) [42–48].

## Discussion

Traditional scientific methods will undoubtedly continue over time to help understand human environments, including environments that are as complex and dynamic as healthcare systems. At the same time, the difficulty of understanding those environments in the concepts and language of sciences suggests that explanatory theories of those environments will be more meaningful when they include contributions from the arts and humanities.

An important, and intriguing, painting by the Belgian surrealist René Magritte hints at the potential of such an ecumenical approach. In this work, Magritte apparently tries to represent the complex, emotionally freighted world of tobacco use by juxtaposing the image of a tobacco pipe with a written comment: “Ceci n’est pas une pipe” (“This is not a pipe”). The resulting cognitive dissonance suggests the artist’s intent is to increase the painting’s impact by cautioning his viewers that “This is only the image of a pipe, not the actual object; don’t confuse the two,” and encouraging them not to mistake the part for the whole (a pipe is, after all, only one small part of tobacco smoking).

But he does not stop there: in his effort to jolt viewers toward even deeper and more precise awareness of tobacco use, Magritte resorts to a particularly unorthodox representation of the pernicious habit, when he flatly asserts that “a pipe actually isn’t a pipe,” his surrogate for a paradoxical characterization of tobacco use in terms of *what it is not*. Examples of this startling apophatic (i.e., reverse) way to represent complex, confusing realities are now appearing in the literature of improvement science, as in “wake-up calls” telling us that *neither* a checklist of infection control measures [49] *nor* a surgical safety checklist  [50], by itself, is an improvement intervention (the unstated subtext being that successful, sustained improvement absolutely requires explicit, extensive coordination, and tight linkage, between the intervention and the environment in which it is being implemented).

In articulating her explanatory theory of the world of *falconry*, the scholar and writer Helen Macdonald also turns, as follows, to this paradoxical, inverse way of understanding the deeper meaning of a complex human environment [51]:

“[T]here is a world of things out there – rocks and trees and grass and all the things that crawl and run and fly. They are all things in themselves, but we make them sensible to us by giving them meanings that shore up our own views of the world. In my time [living with and training my goshawk] Mabel I’ve learned how you feel more human once you have known, even in your imagination, what it is likely to be not”.

## Conclusions

This commentary considers evidence that reinforces the crucial reality that the healthcare systems in which improvement programs take place—or, more specifically, the values and character of those systems—are at least as important in improving care as the specifics of the improvement interventions themselves. This obvious but often underappreciated reality environmental feature argues strongly for the development of sophisticated, nuanced understanding of those environments early in the implementation of improvement programs, and consistent application of that understanding during the improvement process. Realistically, understanding a human environment—especially one as complex and dynamic as a healthcare system—is an arduous, demanding undertaking, which further underscores the value of building a basic set of context-related initiatives into the implementation of any sizeable healthcare improvement program. These initiatives might include the following:

- As early as possible in planning the program, create an explanatory theory of the host environment that incorporates the basic principles of explanation, especially unification of the environment’s major components;
- If possible, involve social scientists, as well as professionals from humanities (e.g., creative writers, reporters, historians, graphic artists and the like) in the development of that explanatory theory;
- Use that explanatory theory in coordinating and linking the intervention with the host environment;
- Explore the use of established mature explanatory theories, individually or collectively, in making sense of the local host environment;
- Assess the relative importance of the environment’s major components as determinants of its nature and behavior; its successes and failures;
- From time to time, review the most current version of the explanatory theory and revise it if necessary as more is learned about the host environment and about the interaction between environment and intervention
- To avoid creating jitter and instability in the program, resist unnecessary tinkering with the makeup and application of the explanatory theory;
- Make explicit efforts to assure that all members of the improvement team are familiar with the major components of the host environment, and understand how those components fit/work together;
- Adapt the focus, comprehensiveness, organization, and level of detail of the explanatory theory of the host environment, to make it as useful as possible for its most important users.

## References

[1] Weick KE, Sutcliffe KM, Obstfeld D (2005). Organizing and the process of sense-making. Organization Sci.

[2] Bate P (2014). Context is everything. Perspectives on context.

[3] Squires JE, Graham ID, Hutchinson AM, Michie S, Francis, JJ, Sales A, et al. Identifying the domains of context important to implementation science: a study protocol. Implement Sci. 2015;10:135. Doi: 10:1186/s13012–015-0325-y. 10.1186/s13012-015-0325-y PMC4584460 26416206

[4] Ogrinc G, Davies L, Goodman D, et al. SQUIRE 2.0 Standards for Quality Improvement Reporting Excellence: revised publication guidelines from a detailed consensus process. BMJ Qual Saf. 2015. 10.1136/bmjqs-2015-004411. 10.1136/bmjqs-2015-004411 PMC5256233 26369893

[5] Vandenbroucke JP (2008). Observational research, randomized trials, and two views of medical science. PLoS Med.

[6] Davidoff F, Dixon-Woods M, Leviton L, Michie S. Demystifying theory and its use in improvement. BMJ Qual Saf 2015;24:228–238. doi 1136/bmjqs-2014-003627. 10.1136/bmjqs-2014-003627 PMC4345989 25616279

[7] Clarke KA, Primo DM (2012). A model discipline. Political science and the logic of representations.

[8] Berwick DM, Godfrey AB, Roessner J (2003). Curing healthcare: new strategies for quality improvement.

[9] Braithwaite, J., Churruca, K., Ellis, LA, et al. Complexity science in healthcare –aspirations, approaches, applications and accomplishments: a white paper. Australian Institute of Health Innovation, Macquarie University: Sydney; 2017; https://www.researchgate.net/publications/319643112. Accessed 1–23-19.

[10] Greenhalgh T (2018). Systems. How to implement evidence-based medicine.

[11] Nardi BA. Studying context: A comparison of activity theory, situated action models, and distributed cognition. In Activity Theory and Human-Computer Interaction. Cambridge: MIT Press; 1995:69-102.

[12] Greenhalgh T, Stones R. Theorising big IT programs in healthcare: Strong structuration theory meets actor-network theory. Soc Sci Med; 2010;70:1285–1294. doi: 10.10.16/j.socscimed.2009.12.034. 10.1016/j.socscimed.2009.12.034 20185218

[13] Pitt JC (1988). Theories of explanation.

[14] Glouberman S, Zimmerman B (2002). Complicated and complex systems: what would successful reform of Medicare look like?.

[15] Hempel CG, Oppenheim P, Pitt JC (1988). Studies in the logic of explanation. Theories of explanation.

[16] Salmon WC, Pitt JC (1988). Statistical explanation and causality. Theories of explanation.

[17] Radford A (1988). Transformational grammar.

[18] Purcell GP, Shortliffe EH. Contextual models of clinical publications for enhancing retrieval from full-text databases. Proc Annu Symp Comput Appl Med Care. 1995:851–7. PMC2579214 8563412

[19] Moskovitch P, Martins SB, Behiri E (2007). A comparative evaluation of full-text, concept-based, and context-sensitive search. J Am Med Inform Assoc.

[20] Jaynes J (1990). The origin of consciousness in the breakdown of the bicameral mind.

[21] Kitcher P. Explanatory Unification, *In* Pitt, reference 11, pp. 167–87*.*

[22] Sheridan TB (2017). Modeling human-system interaction.

[23] Gopnik, A. Rewriting nature. Charles Darwin, natural novelist. The New Yorker, October 23, 2006.

[24] Wears RL, Barsky CL, Perry DJ. Human Factors in Organizational Design and Management. In: Broberg O, Fallentin N, Hasle P, et al., editors. Control modes in care delivery organizations: Nordic Ergonomics Society Annual Conference, XI; 2014. p. 957–6.

[25] Tilly C (2006). Why? What happens when people give reasons…and why.

[26] Schulz L (2015). Infants explore the unexpected. Science.

[27] Weick KE (2009). Making sense of the organization. The impermanent organization. Volume 2.

[28] Daft RI, Weick KE (1984). Toward a model of organizations as interpretation systems. Acad Manag Rev.

[29] Dixon-Woods M, Bosk CL, Aveling EL, et al. Explaining Michigan: developing an ex post theory of an improvement program. Milbank Q. 2011;89:167–205. 10.1111/j.1468-0009.2011.00625.x. https://www.ncbi.nlm.nih.gov/pmc/articles/PMC3142336/. Accessed 12 Mar 2018. 10.1111/j.1468-0009.2011.00625.x PMC3142336 21676020

[30] Pronovost P, Needham D, Berenholtz S (2006). An intervention to decrease catheter-related bloodstream infections in the ICU. N Engl J Med.

[31] Schön DA (1991). The reflective practitioner.

[32] Bradley EH, Curry LA, Cherlin E (2012). Hospital strategies for reducing risk-stratified mortality rates in acute myocardial infarction. Ann Intern Med.

[33] Kaplan HC, Provost LP, Froehle CM (2012). The model for understanding success in quality (MUSIQ): building a theory of context in healthcare quality improvement. BMJ Qual Saf.

[34] Damschroder LJ, Aron DC, Keith RE (2009). Fostering implementation of health services research findings into practice: a consolidated framework for advancing implementation science. Implement Sci.

[35] Taylor SL, Dy S, Foy R (2011). What context features might be important determinants of the effectiveness of patient safety practice interventions?. BMJ Qual Saf.

[36] Gustafson DH, Sainfort F, Eichler M (2003). Developing and testing a model to predict outcomes of organizational change. Health Serv Res.

[37] Bate P, Mendel P, Robert G (2008). Organizing for quality.

[38] Greenhalgh T, Stones R. Theorizing big IT programs in healthcare: strong structuration theory meets actor-network theory. Soc Sci Med*.* 2010;70:1285–1294. doi 0.10.16/j.socscimed. 2009.12.034. 10.1016/j.socscimed.2009.12.034 20185218

[39] Bernstein L. The unanswered question: whither music? Cambridge, MA: Harvard University Press; 1981.

[40] Ammenwerth E, Iller C, Mahler C (2006). IT-adoption and the interactions of task, technology, and individuals: a fit framework and a case study. BMC Med Inform Decis Mak.

[41] Lesselroth BJ, Yang J, McComachie J (2011). Addressing the sociotechnical drivers of quality improvement: a case study of post-operative DVT prophylaxis computerized decision support. BMJ Qual Saf.

[42] Hawe P, Reilly T (2005). Ecological theory in practice. Illustrations from a community-based intervention to promote the health of recent mothers. Prev Sci.

[43] Lanham HJ, McDaniel RR, Crabtree BF, Miller WL, Stange KC, Talka AF, et al. How improving practice relationships among clinicians and nonclinicians can improve quality in primary care. Jt Comm J Qual Patient Saf 2009;35:457–456; PMCID:2928073. 10.1016/s1553-7250(09)35064-3 PMC2928073 19769206

[44] Leykum LK, Pugh J, Lawrence V, et al. Organizational interventions employing principles of complexity science have improved outcomes for patients with type II diabetes. Implementation Sci. 2007;2(28). 10.1186/1748-5908-2-28. 10.1186/1748-5908-2-28 PMC2018702 17725834

[45] Leykum LK, Parchman M, Pugh J (2010). The importance of organizational characteristics in patients with chronic disease: a systematic review of congestive heart failure. Implementation Sci.

[46] Hawe P, Shiell A, Riley T (2009). Theorizing events in systems. Am J Community Psychol.

[47] Leykum LK, Lanham JH, Provost SM (2014). Improving outcomes of hospitalized patients: the physician relationships, improvising, and sense making protocol. Implementation Sci.

[48] McAllister C, Leykum LK, Lanham H (2014). Relationships within impatient physician housestaff teams and their association with hospitalized patient outcomes. J Hosp Medicine.

[49] Bosk CL, Dixon-Woods M, Goeschel CA, Pronovost PJ. Reality check for checklists. Lancet 2009;374:444–445. PMID:19681190. 10.1016/s0140-6736(09)61440-9 19681190

[50] Aveling EL, McCulloch P, Dixon-Woods M. A qualitative study comparing experiences of the surgical safety checklist in hospitals in high-income and low-income countries. BMJ Open. 2013:e003039. 10.1136/bmjopen-2013-003039. 10.1136/bmjopen-2013-003039 PMC3752057 23950205

[51] Macdonald H (2014). H is for hawk.

